# Dietary Protein Optimization for Growth and Immune Enhancement in Juvenile Hybrid Sturgeon (*Acipenser baerii* × *A. schrenckii*): Balancing Growth Performance, Serum Biochemistry, and Expression of Immune-Related Genes

**DOI:** 10.3390/biology13050324

**Published:** 2024-05-06

**Authors:** Chang’an Wang, Entong Liu, Hui Zhang, Honghe Shi, Guangwen Qiu, Shaoxia Lu, Shicheng Han, Haibo Jiang, Hongbai Liu

**Affiliations:** 1 Key Laboratory of Aquatic Animal Diseases and Immune Technology of Heilongjiang Province, Heilongjiang River Fisheries Research Institute, Chinese Academy of Fishery Sciences, Harbin 150070, China; wangchangan@hrfri.ac.cn (C.W.);; 2 College of Animal Science and Technology, Northeast Agricultural University, Harbin 150030, China; 3 College of Animal Science, Guizhou University, Guiyang 550000, China

**Keywords:** hybrid sturgeon, dietary protein, growth performance, immune response, gene expression

## Abstract

**Simple Summary:**

This research assessed the effects of dietary protein levels on juvenile hybrid sturgeon growth and health over 12 weeks, with protein levels varying from 30% to 45%. It identified an optimal protein range of 35.9% to 38.3%, which maximized weight gain, improved protein efficiency, and enhanced serum health indicators, including essential amino acid content. Immune system performance also peaked within this range, correlating with increased activity of growth and immune-related genes in the intestine, indicating diet–gene interaction. Exceeding this range showed no additional benefits and could harm the fish. These findings underline the necessity of fine-tuning dietary protein for optimal aquaculture outcomes in hybrid sturgeon.

**Abstract:**

This study aimed to evaluate the effects of dietary protein levels on growth performance, serum indices, body amino acid composition, and intestinal gene expression in juvenile hybrid sturgeon (*Acipenser baerii* × *A. schrenckii*). Hybrid sturgeons (initial weight 29.21 ± 2.04 g) were fed isolipidic diets containing 30%, 33%, 36%, 39%, 42% or 45% crude protein for 12 weeks (*n* = 18 tanks, 30 fish/tank). Results showed significant differences between treatments, where weight gain and protein efficiency ratio peaked optimally between 35.9% and 38.3% dietary protein. Serum parameters such as glucose, alanine aminotransferase, aspartate aminotransferase, superoxide dismutase, and lipid peroxidation levels varied significantly with changes in dietary protein levels. Specifically, the highest enzymatic activities and growth parameters were observed in groups fed with 33% to 39% protein, enhancing whole-body concentrations of lysine, leucine, phenylalanine, proline, and glutamic acid. Immune parameters such as immunoglobulin M and lysozyme activity also showed peak levels at higher protein concentrations, particularly notable at 42% for lysozyme and 36% for both component 3 and immunoglobulin M. Gene expression related to immune and growth pathways, including MyD88, TLR1, IL-8, IL-6, NF-κB, and IL1β, was significantly upregulated at protein levels of 33% to 36%, with a noted peak in expression at 39% for TLR1, IL-10, and TOR signaling genes, before diminishing at higher protein levels. Overall, the dietary protein requirement for juvenile hybrid sturgeon ranges from 35.9% to 38.3% crude protein.

## 1. Introduction

Sturgeons, belonging to one of the oldest groups of cartilaginous, hard-scaled fish, play a significant role in aquaculture due to their economic importance. The hybridization of *Acipenser schrenckii*, native to the Amur River Basin, with *Acipenser baerii*, known for its disease resistance but slower growth rate, has yielded a vigorous, fast-growing, and highly disease-resistant offspring. This hybrid (*Acipenser baerii* × *A. schrenckii*), successfully produced around 2007, represents a milestone in sturgeon aquaculture, combining rapid growth with resilience, thereby setting new benchmarks for production and sale scales in China [1,2]. Despite the economic importance of hybrid sturgeons, research into their specific nutritional needs, especially protein requirements, is scant, highlighting a gap in aquaculture science that this study seeks to address.

Protein is foundational to fish aquaculture, serving as the bedrock for growth, metabolic stability, and overall health by providing essential amino acids for tissue regeneration and the synthesis of vital biomolecules like enzymes and antibodies. As it directly influences biomass increase in fish, dietary protein underpins the significance of optimized protein intake for aquaculture sustainability [3,4]. The balance in dietary protein levels is a delicate one: insufficient amounts curtail growth and impair health, whereas excess leads to inefficient energy conversion and heightened environmental burden through increased ammonia excretion [5].

Historical dietary research on sturgeons has covered various nutrients, such as proteins, lipids, vitamins, minerals, bioactive compounds like caffeic acid [6], tannins [7], and sodium propionate [8], demonstrating the complexity of their nutritional ecology. Past studies across various sturgeon species have identified an optimal dietary protein range of about 40%, thus highlighting the importance of species-specific research to accurately determine their precise nutritional needs [9,10,11,12].

The immune system’s functionality in fish, particularly how it responds to dietary protein levels, has garnered attention, with findings indicating that optimal protein intake can significantly enhance immune markers such as lysozyme activity, complement components, and immunoglobulin levels [13]. This study further explores the genetic underpinnings of dietary protein’s effects, examining gene expression within the endocrine systems that regulate growth and immune and nutritional status, notably the Toll-like receptor (TLR), GH–IGF axis, and the TOR signaling pathway, which are pivotal in understanding fish growth dynamics [14].

Thus, this investigation is poised to elucidate the optimal dietary protein requirements for hybrid sturgeons, aiming to provide a cornerstone for the development of feeds that are not only nutritionally balanced but also cost-efficient and environmentally benign. By delving into the interplay between dietary protein levels, growth performance, immune system bolstering, and gene expression, this research contributes a vital piece to the puzzle of sustainable aquaculture practices, ensuring the health and profitability of hybrid sturgeon farming.

## 2. Materials and Methods

### 2.1. Fish Management

Hybrid sturgeon juveniles (initial weight: 29.21 ± 2.04 g) were acquired from the Fangshan Sturgeon Breeding Station, Chinese Academy of Fishery Sciences. At the beginning of the study, the sturgeons were acclimatized to the experimental environment for 14 days. Sturgeons were randomly separated into 18 tanks (300 L each; 30 fish/tank). Water quality parameters were monitored once a day. During the 12-week trial, the tanks were maintained under a relatively constant dissolved oxygen level (5.8–6.0 mg/L), temperature (22.5–23.5 °C), pH (7.8–8.0), ammoniacal nitrogen level (<0.02 mg/L), and photoperiod (12 h light: 12 h dark). The fish were fed four times daily (6:00, 10:00, 14:00, and 18:00) until apparent satiety.

### 2.2. Experimental Diets

Six isolipidic feeds were used, with protein levels of 30% (G1), 33% (G2), 36% (G3), 39% (G4), 42% (G5), and 45% (G6). Casein, fishmeal and wheat gluten were utilized as protein sources, dextrin, and wheat middlings as carbohydrate sources, phospholipids, fish oil, and soybean oil as lipid sources (Table 1). The diets were passed through a 250 µm sieve. The dry, finely ground feed components were completely blended with lipids and then homogenized using a blender (GYJ-250B; Dashiqiao Bao’s Feed Machinery Factory). The resulting dough was pelleted into 1.5 mm (diameter) granular feed using a lab pelletizer (SLP-45; Fishery Mechanical Facility Research Institute, Shanghai, China). The pellets were then air-dried to a moisture content of approximately 10% by weight, gathered in vacuum-packed bags, and stored at −20 °C until use.

### 2.3. Sample Collection

The fish were fasted for 24 h before sampling. For this experiment, fish were anesthetized using 60 mg/L of MS-222 (tricaine methanesulfonate, Sigma-Aldrich, St. Louis, MO, USA). From each tank, four fish were selected for analysis of body composition, evaluation of amino acid profile, and examination of blood chemistry. Additionally, three fish from each tank were chosen for gene expression analysis of the mid-intestine (the section following the pyloric caecum). Blood samples were drawn from each fish’s tail vein and then maintained at 4 °C for 4 h to allow serum separation. Subsequently, the samples were centrifuged at 5000× *g* for 15 min in a precooled centrifuge. The supernatant was transferred to a new tube and stored at −80 °C.

Following blood collection, fish were humanely euthanized with an overdose of MS-222 (200 mg/L, Sigma-Aldrich, St. Louis, MO, USA), ensuring rapid and painless euthanasia. This allowed for the subsequent collection of tissues without distressing the specimens. The digestive tracts were flushed with physiological saline to clear any contents, and excess lipids and inclusions were removed from the mid-intestines. Tissues designated for gene expression analysis were immediately placed in cryovials and submerged in liquid nitrogen for RNA extraction.

For comprehensive analyses, whole bodies from each treatment group were labeled and stored at −80 °C for subsequent determination of body composition and whole-body amino acid profiles.

### 2.4. Nutrient Content

The experimental diets and whole fish were analyzed following the AOAC protocols (2016) [15]. The moisture content of the samples was analyzed using the direct drying method. The samples were put in an oven at 105 ± 0.5 °C for 3 h until they reached a constant weight. The protein content of the whole fish was determined using automatic rapid nitrogen fixing (rapid N exceed, Elementar, Langenselbold, Germany). The ash content was determined by complete combustion of samples. Each sample (2.0 ± 0.5 g) was then ignited in the muffle furnace to ensure thorough combustion (600 °C, 2 h). The crude lipid content was determined using the Soxhlet extraction method with a Soxhlet extraction system (Extraction system-811, BUCHI, Flawil, Switzerland) and petroleum ether as the extraction solvent. The extraction process lasted for 10 h to ensure complete extraction of the lipid content from the sample. The gross energy content was determined through the utilization of a bomb calorimeter (LRY-600A, produced by Chuangxin Ltd., Hebei, China).

### 2.5. Amino Acid Determination

An automated amino acid analyzer (L-8900, Hitachi, Ibaraki, Japan) was fully utilized to determine the amino acid composition of fish [16]. Whole fish and feed powder samples (40–50 mg) underwent hydrolysis with the use of hydrochloric acid (10 mL of 6 mol/L), and the sample hydrolysate (110 ± 1 °C for hydrolysis for 22 h) was obtained after evaporation (60 °C) and washing treatment. Both the sample hydrolysate and the standard solution were then injected into the auto-sampling bottle, followed by the detection of amino acid content through the amino acid analyzer.

### 2.6. Serum Biochemical Analysis

To analyze serum antioxidant and immune indicators, biochemical analyses were performed, with enzyme activities and other biochemical parameters expressed per milliliter or liter, for superoxide dismutase (SOD), malondialdehyde (MDA), alkaline phosphatase (ACP), lysozyme (LZM), and immunoglobulin M (IgM) following the manufacturer’s protocol utilizing commercially available kits from the Nanjing Jiancheng Bioengineering Institute, Nanjing, China (Table 2).

The serum biochemical parameters alanine aminotransferase (ALT), aspartate aminotransferase (AST), triglycerides (TG), total protein (TP), glucose (GLU), and complement components C3 and C4 were quantitatively analyzed using an MNCHIP Celercare V5 automatic biochemical analyzer (MNCHIP, Shanghai, China).

### 2.7. Quantitative Real-Time Polymerase Chain Reaction (qRT-PCR)

The intestinal tissue samples were collected, treated with an appropriate amount of liquid nitrogen, and ground in a cooled mortar maintained at −80 °C until they were fine powder. Total RNA was extracted from the powder using TRIzol reagent (Ambion, San Diego, CA, USA). To remove contaminating DNA, the sample was treated with Rnase-free Dnase I (Thermo Scientific, Waltham, MA, USA). The mass of RNA was measured by agarose gel electrophoresis, for which a 1% agarose gel was prepared and run at 110 V for 30 min. The concentration and quality of the extracted RNA required for subsequent experiments were determined using an ultra-micro UV-visible spectrophotometer (Thermo Scientific NanoDrop 2000) with an A260 nm/A280 nm ratio between 1.8 and 2.0. Reverse transcription was performed using the PrimeScriptTM RT Reagent Kit (Takara, Dalian, China) according to the kit instructions. The cDNA concentration was measured using an ultra-micro spectrophotometer, and samples with completed reverse transcription were diluted to 50 ng/μL for dispensing and storage.

Use the NCBI website to design primers, ensuring avoidance of repeated bases and adherence to a melting curve Tm value of 55–80 °C. It is typically recommended that primer length falls between 18 and 27 bp, but should not exceed 38 bp, with a primer GC content of 30–80%. The length of the PCR amplification product should fall within the range of 80–250 bp, with a primer annealing temperature of around 60 °C. Once primer design is complete, a BLAST test should be carried out to verify its specificity (Table 3).

RT-PCR was performed using the Takara TB Green^®^ Premix Ex Taq^TM^ II (Tli RnaseH Plus) Kit, LightCycler^®^ 480 Real-Time PCR System. β-actin was used as housekeeping gene. Real-time PCR assays were performed on an ABI 7500 Real-Time instrument (Applied Biosystems, Waltham, MA, USA). Three replicates of each amplification reaction were used for comparison (Table 4). Relative gene expression level was determined by the 2^−∆∆CT^ method [17].

### 2.8. Calculations and Statistical Analysis

Survival rate (SR, %) = (total fish harvested/total fish cultured) × 100%; weight gain rate (WGR, %) = ((W_1_ − W_2_)/W_2_) × 100%; specific growth rate (SGR, %/d) = (ln(W_1_/W_2_)/D) × 100%; protein efficiency ratio (PER, %) = ((W_1_ − W_2_)/protein intake (g)) × 100%; feed conversion ratio (FCR) = W_3_/total biomass gain (g); hepatosomatic index (HIS, %) = (W_4_/W_1_) × 100%; where W_1_, W_2_, and D mean the wet fish weight (g), initial fish weight (g), and feeding period (d), respectively, and W_3_ and W_4_ mean the dry feed intake (g), and liver weight (g), respectively.

Data in the present study were statistically analyzed with 22.0 (SPSS, Chicago, IL, USA), and all data were tested using Levene’s equal variance test for homogeneity of variance. One-way ANOVA was used to assess the statistical significance among groups, and mean separations were established by using Duncan’s multiple range test. Plots were generated using Origin 2017 (OriginLab, Northampton, MA, USA), while graphs were created with GraphPad Prism version 9.0 (GraphPad Software, San Diego, CA, USA). The second-order polynomial regressive model and broken-line model were used to assess the optimal dietary protein levels. Data are expressed as means ± S.E., and differences were deemed to be statistically significant at *p* < 0.05.

## 3. Results

### 3.1. Growth

**Figure 1:**
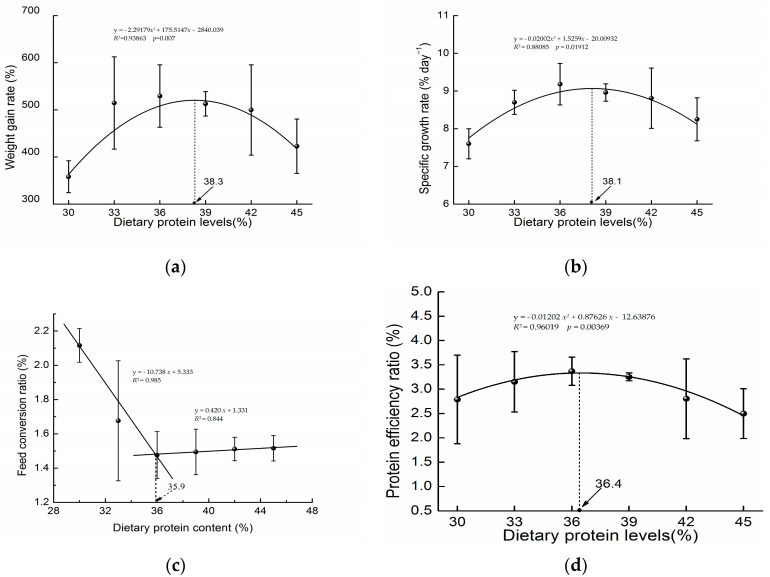
Analysis of optimal dietary protein levels for growth and feed efficiency in hybrid sturgeon through models. (**a**) Quadratic curve analysis of the relationship between dietary protein levels and weight gain rate; (**b**) quadratic curve analysis of the relationship between dietary protein levels and specific growth rate; (**c**) broken-line analysis of the relationship between dietary protein levels and feed conversion ratio; (**d**) quadratic curve analysis of the relationship between dietary protein levels and protein efficiency ratio. The data are reported as means ± error (*n* = 3).

The growth performances of juvenile hybrid sturgeon-fed diets with various protein levels are shown in Table 5. Fish SR ranged from 81.33% to 92.00%. Dietary protein content significantly affected WGR, FCR, SGR, PER, and HSI (*p* < 0.05). The relationships of dietary protein content with WGR, SGR, and PER were demonstrated using quadratic curve models, which revealed that the optimal range of dietary protein for the maximum growth performance of hybrid sturgeon was 36.4–38.3%. On the foundation of the broken-line model assay with FCR as the appraisal standard, the optimal dietary protein level of the diet for the maximum growth performance was 35.9% (Figure 1). The hybrid sturgeon fed the 39% diet exhibited remarkably greater HSI than those fed the 36%, 42%, and 45% protein diets (*p* < 0.05).

### 3.2. Body Composition

Dietary protein levels significantly affected the moisture, crude protein, ash, and crude lipid contents in the whole body (*p* < 0.05, Table 6). The hybrid sturgeon in the 42% protein group yielded significantly more crude protein than the fish in the 30% protein groups (*p* < 0.05), and the fish in the 30% and 45% protein groups had significantly lower ash content than those in the other treatment groups (*p* < 0.05). The hybrid sturgeon in the 33% protein group yielded significantly lower moisture contents than other protein groups. Crude lipid contents initially increased with dietary protein levels from 30% to 42%, peaking at 13.31%, then slightly decreased at 45% protein. This indicates an optimal dietary protein range of 39% to 42% for protein and lipid accumulation.

### 3.3. Amino Acids Profile

The dietary protein levels significantly affected valine, leucine, phenylalanine, lysine, isoleucine, proline, and glutamic acid contents (Table 7; *p* < 0.05). The hybrid sturgeon in the 33%, 36%, and 39% protein groups yielded significantly more leucine, lysine, and proline than the fish in the 30% protein group (*p* < 0.05), and the fish fed the 39%, 42% and 45% diet yielded significantly more glutamic acid and isoleucine than sturgeon fed the 30% diet (*p* < 0.05). However, the phenylalanine contents of hybrid sturgeon fed the 30%, 33%, 36%, and 39% protein diets were similar.

### 3.4. Plasma Metabolite Contents

The TG, TP, ALT, AST, GLU, SOD, and MDA levels of juvenile hybrid sturgeon fed a variety of dietary protein levels are shown in Table 8. The effects of dietary protein levels on GLU, ALT, TG, SOD, and MDA levels were significant (*p* < 0.05). Specifically, G3 and G4 exhibited significantly higher GLU levels than G1 (*p* < 0.05), marking a clear influence of dietary variations on glucose metabolism. Similarly, G2 and G3 showed elevated levels of ALT, indicating changes in liver enzyme activities due to dietary protein levels, with G2 displaying significantly higher ALT levels than G1, G5, and G6 (*p* < 0.05). The TG levels in G3 were significantly higher than those in G1 (*p* < 0.05), reflecting differences in lipid metabolism among the groups. Moreover, a remarkable increase in SOD activity was observed in G3–G6 compared to G1 (*p* < 0.05), suggesting an enhanced antioxidative defense mechanism in response to specific dietary proteins. However, TP and ALB levels across all groups remained unaffected by the dietary protein levels (*p* > 0.05). MDA levels showed significant variations, with G2–G6 exhibiting lower levels (*p* < 0.05) than G1.

### 3.5. Hematological Immune Parameters

Plasma immune parameters are shown in Table 9. LZM activity significantly increased from 99.46 ± 10.34 U/mL in the 30% protein group to 287.53 ± 11.99 U/mL in the 42% protein group (*p* < 0.05). ACP levels showed a substantial elevation from 257.97 ± 6.57 U/mL in the 30% protein group to 370.82 ± 3.95 U/mL in the 39% protein group (*p* < 0.05). C3 and C4 levels were significantly enhanced, with the highest levels observed in the 36% protein group for C3 (16.41 ± 0.56 mg/mL) and in the 42% protein group for C4 (1.75 ± 0.16 mg/mL) (*p* < 0.05). IgM concentrations increased with dietary protein, peaking at 118.90 ± 8.55 mg/mL in the 36% protein group (*p* < 0.05).

### 3.6. Gene Expression Profiling

**Figure 2:**
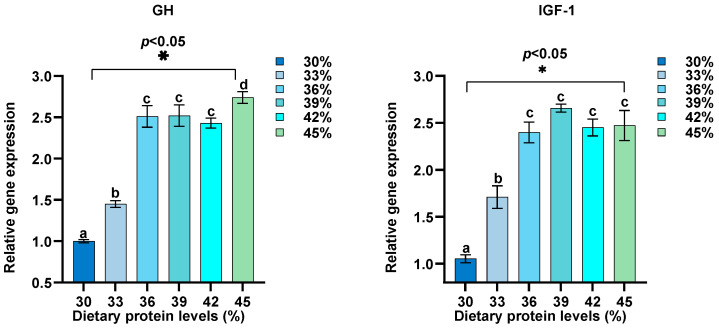
Effects of different protein levels on GH and IGF-1 expression in the intestine of hybrid sturgeon (*n* = 3). β-actin was used as the housekeeping gene to normalize the data. Letters indicate significant differences.

**Figure 3:**
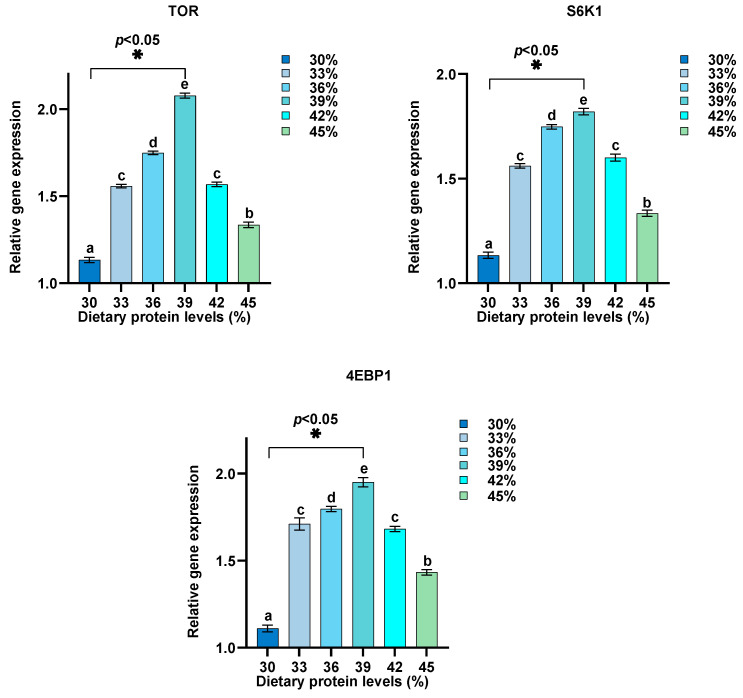
Impact of varied protein levels on the expression of TOR, S6K1, and 4EBP1 in the intestine of hybrid sturgeon (*n* = 3). β-actin was used as the housekeeping gene to normalize the data. Letters indicate significant differences.

**Figure 4:**
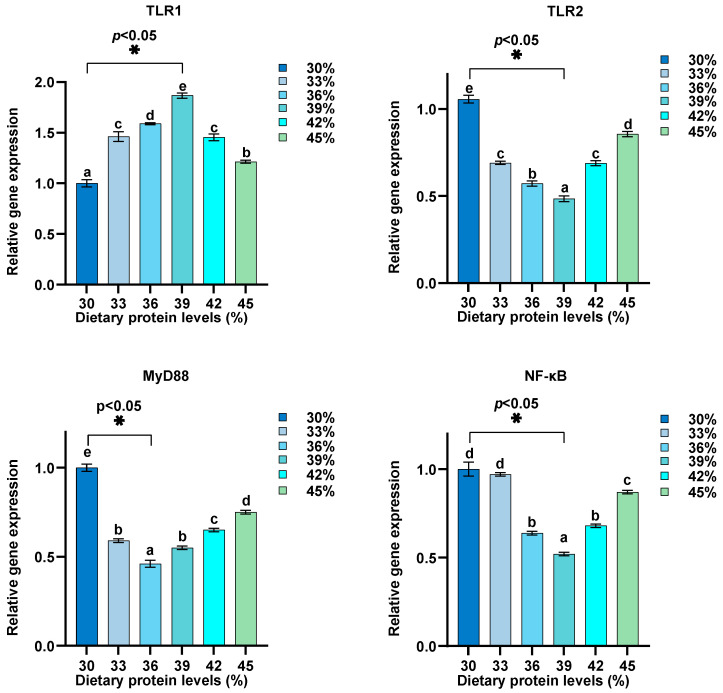
Influence of dietary protein levels on innate immunity gene expression in the intestine of hybrid sturgeon (*n* = 3). β-actin was used as the housekeeping gene to normalize the data. Letters indicate significant differences.

**Figure 5:**
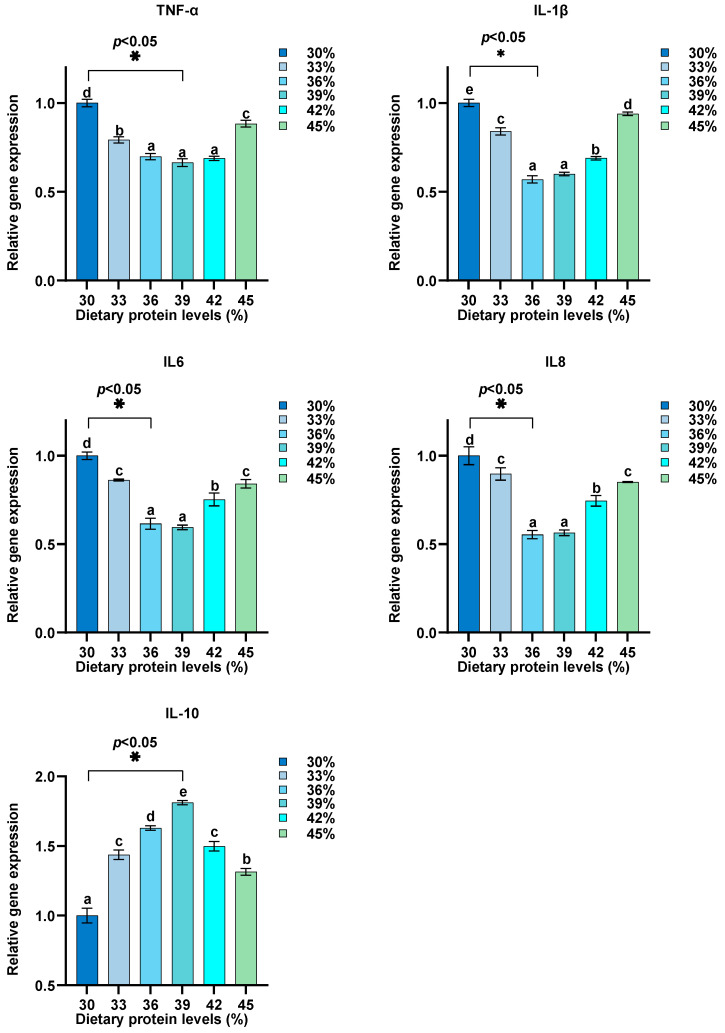
Effects of dietary protein levels on inflammatory response gene expression in the intestine of hybrid sturgeon (*n* = 3). β-actin was used as the housekeeping gene to normalize the data. Letters indicate significant differences.

Dietary protein levels were adjusted between 30% and 45%, and their effects on the relative expression of GH, IGF-1, MyD88, TLR1, IL-8, IL-6, NF-κB, IL1β, TNF-α, IL-10, TLR2, TOR, 4EBP1, and S6K1 in intestine were assessed (Figure 2, Figure 3, Figure 4 and Figure 5). Our findings reveal a distinctive pattern of gene expression modulation in response to dietary protein alterations. At dietary protein levels of 33% and 36%, a notable modulation in the expression of MyD88, TLR1, IL-8, IL-6, NF-κB, and IL1β was observed, indicating a peak in immune responsiveness. Specifically, the expression levels of TLR1, IL-10, GH, IGF-1, and TOR-related genes demonstrated significant upregulation at higher protein levels, peaking at 39%, before displaying a gradual decrease at 45%. Conversely, genes such as TNF-α and TLR2 exhibited a downregulation with increasing protein levels, indicating a potential suppressive effect of high dietary protein on certain immune pathways. The GH/IGF-1 and TOR signaling pathway, through its components GH, IGF-1, TOR, 4EBP1, and S6K1, showed enhanced activation at protein levels up to 39%, beyond which a diminishing response was evident.

## 4. Discussion

### 4.1. Growth Performance

This investigation into the dietary protein requirements and feed utilization efficiency of hybrid sturgeons showed considerable protein demand (35.9–38.3%). This finding is in agreement with research on various other fish species, including *Diplodus vulgaris* [18], *Acanthopagrus schlegelii* [19], *Clarias magur* [20], and *Macrobrachium nipponense* [21], all of which have shown similar dietary protein requirements. Consistent with the literature, protein deficiency is often a limiting factor for fish growth, as higher dietary protein levels are known to facilitate more rapid growth [22,23]. Nonetheless, the adverse effects of protein levels exceeding the optimal range, including compromised protein synthesis capacity [24] and decreased energy utilization [25], underscore the importance of determining an ideal protein-to-energy ratio to optimize growth performance in sturgeon aquaculture.

Moreover, the feed conversion ratio (FCR) serves as a critical indicator of aquaculture efficiency, reflecting the effectiveness of converting feed into biomass [26]. The observed trend in FCR, where it decreased with increasing dietary protein levels up to 36%, highlights the optimal utilization of dietary protein for growth at this percentage. Beyond 36% protein, it appears that additional protein is primarily utilized for energy rather than growth, suggesting diminishing returns on growth efficiency. This finding is consistent with data from studies on other fish species, such as hybrid sturgeon *Acipenser baerii* × *A. gueldenstaedtii* [11], *Rhamdia quelen* [27], *Trachinotus blochii* [28], and *Oreochromis niloticus* [29], which also reported improved FCR with higher dietary protein levels up to an optimal point. Given that the diets were isoenergetic, it is unlikely that energy availability influenced feed intake. Instead, increased protein beyond 36–39% likely exceeded the daily protein requirement for muscle accretion, as evidenced by the PER findings, and was utilized for energy. This shift in protein utilization underscores the importance of balancing dietary protein to meet but not excessively surpass the growth-specific requirements of hybrid sturgeon. Notably, the dynamics of higher protein intake might also enhance satiety, potentially influencing overall energy intake and growth, thus adding complexity to dietary formulations in aquaculture [30]. Contrary to the findings on *O. niloticus* [31] and *Nibea coibor* [32], which reported an initial FCR increase followed by a decrease with higher protein content, our study demonstrates the nuanced impact of dietary protein on FCR in hybrid sturgeons. This highlights the species-specific nature of dietary requirements and the potential for tailored nutritional strategies to improve aquaculture productivity.

### 4.2. Body Amino Acids

Our study elucidates the pivotal role of dietary protein levels in modulating the amino acid profiles in hybrid sturgeon, demonstrating marked increases in essential amino acids such as lysine, leucine, and isoleucine with higher-protein diets. These findings echo those observed in sterlet sturgeon (*Acipenser ruthenus*), highlighting the universal importance of balanced dietary proteins for optimal fish growth and health through enhanced amino acid assimilation [33]. Furthermore, the nonlinear response of phenylalanine suggests a potential species-specific adaptation or saturation threshold in amino acid metabolism, a phenomenon also noted in other piscine species [34].

While increases in amino acids correlate with dietary protein levels, the direct link between these elevated amino acid concentrations and enhanced biological functionality or growth performance is not straightforward. The presence of higher amino acid levels does not automatically translate to improved health or growth, underscoring the necessity for a balanced approach considering both amino acid availability and digestibility [35].

Although our study did not directly analyze amino acid levels in serum, understanding their bioavailability and metabolic utilization through dietary interventions is crucial. Reflecting immediate dietary intake, plasma amino acid concentrations can indicate metabolic responses to diet composition, as demonstrated in other species [36]. Our analysis of dietary and body amino acid profiles revealed differential uptake and utilization, where essential amino acids such as lysine and leucine showed effective assimilation, unlike phenylalanine, which exhibited little variation across diets.

These differential responses to dietary amino acids underscore the complex interplay among diet, genetics, and metabolic regulation, suggesting that while dietary modifications can enhance nutrient availability, genetic factors largely govern protein synthesis in fish, determining the efficiency and pattern of synthesis irrespective of dietary abundance [37].

To further clarify the impact of dietary amino acids on serum profiles and overall metabolic health in hybrid sturgeon, future research should include detailed analyses of blood or serum post-dietary interventions. Such studies are essential for elucidating amino acid metabolism kinetics and their endocrine regulation, crucial for tailoring dietary formulations to optimize growth and health in aquaculture settings [38].

### 4.3. Plasma Metabolites

Total protein (TP), comprising both albumin and globulin, serves as an indicator of overall protein synthesis and nutritional status in fish, reflecting their health and well-being [39]. Anomalies in TP levels necessitate detailed analyses to discern which specific protein fraction is impacted, underscoring the need for precise nutritional management. However, in the current study, TP levels remained unaffected by dietary protein variations, presenting a contradiction to findings in *Acanthopagrus schlegelii*, where TP levels exhibited a direct correlation with dietary protein intake [40]. This discrepancy could be attributed to differences in species-specific metabolic responses or dietary formulations. For instance, in juvenile tiger puffer (*Takifugu rubripes*), TP levels were positively associated not with dietary protein but with lipid levels, suggesting a complex interaction between dietary macronutrients in influencing TP [41]. The isolipidic nature of diets in the present study could potentially explain the lack of significant findings regarding TP levels. Additionally, it is important to consider that the blood profiles in our study were collected from fish that had been starved for 24 h, which may have influenced the similarity in TP levels across different dietary treatments. Fasting could potentially normalize some blood parameters, masking variations that might otherwise reflect differences in dietary protein intake.

Enzymatic agents, specifically alanine aminotransferase (ALT) and aspartate aminotransferase (AST), are fundamental to the processes involved in nitrogen metabolism, essential for the synthesis of proteins and for supplying energy in the liver, thus acting as biomarkers for nutritional and health status in fish [42]. The positive correlation observed between dietary protein and activities of ALT and AST in this study is consistent with previous research on *Procambarus clarkii* and *Sparus macrocephalus*, indicating an adaptive mechanism in hybrid sturgeons to modulate protein catabolism following adjustments in levels of protein intake [43,44]. Conversely, in *Larimichthys crocea*, dietary protein did not significantly alter liver ALT or AST activity, likely reflecting its efficient protein digestion and utilization capabilities [45]. This indicates species-specific variations in the response to dietary protein, with certain species like *Aristichthys nobilis* experiencing potential liver damage at high dietary protein levels, as indicated by increased blood AST and ALT levels [46].

Plasma glucose (GLU) levels serve as an additional indicator of dietary protein utilization, reflecting the intricate balance among intake, hepatic synthesis, and cellular uptake processes, which include glycolysis, gluconeogenesis, and glycogenesis [47]. Furthermore, the activities of ALT and AST are intricately linked to GLU production through the mobilization of amino acids via gluconeogenesis [48]. The elevation of AST and GLU levels in fish fed a 36% protein diet in this study could indicate mobilization of aspartate for GLU production, aligning with observations in *O. niloticus*, where increased GLU levels were associated with improved growth and development, underpinning the crucial role of dietary protein in supporting energy demands [49].

The investigation into the impact of dietary protein on antioxidant capability, such as superoxide dismutase (SOD) activity and malondialdehyde (MDA) levels, highlighted the complex relationship between nutrition, immune function, and oxidative stress management in fish [50]. Despite the lack of significant changes in SOD activity and MDA levels with varying dietary protein levels, contrasting findings in *Epinephelus coioides* and *O. niloticus* suggest a potential positive correlation between dietary protein and antioxidant capacity [51,52]. These differences may reflect the multifaceted nature of the interactions between dietary components, immune status, and the body’s capacity to neutralize reactive oxygen species, which revealed notably increased levels of SOD and MDA with dietary protein increased in this study.

### 4.4. Immune Response

The observed increases in LZM activity, ACP levels, and C3 and C4 complement components with higher dietary protein percentages underscore the critical role of protein in enhancing the innate immune system of juvenile hybrid sturgeon. This is consistent with existing literature suggesting that adequate protein nutrition is vital for optimal immune responses in fish [53]. Specifically, the marked elevation in LZM and ACP activities in groups receiving higher-protein diets indicates a strengthened first-line defense against pathogens, likely due to the provision of essential amino acids for synthesizing these immune molecules [54]. Furthermore, the significant enhancements in C3 and C4 levels, particularly in groups fed with 36% and 42% protein, align with findings that complement proteins play a key role in the opsonization and clearance of pathogens, enhancing the fish’s disease resistance [55]. The relationship between dietary protein levels and IgM concentrations, peaking in the 36% protein group, corroborates the notion that dietary nutrients directly influence the adaptive immune system, impacting antibody production and overall immunocompetence [56]. These findings contribute to the growing body of evidence supporting the optimization of dietary protein levels for improving sturgeon health and disease resistance. However, the results also highlight the need for a balanced approach to dietary formulation, as excessive protein can lead to environmental and metabolic inefficiencies [4].

### 4.5. Gene Expression

Growth hormone (GH) and insulin-like growth factor 1 (IGF-1) are crucial in managing nutrient metabolism processes and are fundamental in stimulating overall body development across vertebrates, including fish [57]. GH operates by binding to its specific receptors located in various target tissues, subsequently initiating the production of IGF-1 within the liver and peripheral tissues. IGF-1 is a critical mediator of the growth-promoting effects attributed to GH, influencing numerous physiological processes essential for growth and development [58]. The correlation between hepatic IGF-1 mRNA levels and growth rates in fish underscores the importance of this hormonal axis in aquaculture species [59]. Our findings indicate that dietary protein levels significantly influence the expression levels of GH and IGF-1 in juvenile hybrid sturgeons, with higher dietary protein content correlating with an upregulation in hepatic GH and IGF-1 expression. This pattern is consistent with observations in other fish species, such as *Larimichthys polyactis* [22] and hybrid striped bass (*Morone saxatilis* × *M. chrysops*) [60], highlighting the conserved nature of this growth-regulatory mechanism across different taxa. However, the literature also presents instances where increased dietary protein led to a reduction in IGF-1 levels, as documented by Gomes et al. (2003) [61]. This apparent contradiction might be attributed to the multifaceted nature of hormonal regulation, which can be influenced by myriad factors, including environmental temperature, the metabolic state of the organism, and the complex interplay between different hormones [62].

This study on juvenile hybrid sturgeon has shown that dietary protein levels also significantly influence the expression of genes associated with immune function. Modulation of gene expression was particularly noted at dietary protein levels of 33% and 36%, highlighting an enhanced immune responsiveness through upregulated expression of MyD88, TLR1, IL-8, IL-6, NF-κB, and IL1β. This suggests that specific dietary protein levels can optimize immune gene expression, with peak responses observed at 39% protein before a reduction at 45%. Such findings align with the understanding that dietary nutrients play an important role in modulating immune responses in fish [63]. However, it is important to clarify that changes in gene expression may not directly reflect alterations in protein activity. The relationship between diet, gene expression, and protein function should be interpreted cautiously, as the actual protein activities were not measured in this study. The observed gene expression levels indicate potential impacts on immune function, but their actual effects at the protein level and subsequent physiological responses remain putative without further protein activity analysis.

The downregulation of genes such as TNF-α and TLR2 with increased protein levels indicates a nuanced relationship between dietary protein and immune pathway activation, potentially suggesting a suppressive effect of high dietary protein on specific immune responses. Meanwhile, the enhanced activation of the GH/IGF-1 and TOR signaling pathways up to a protein level of 39% underscores the importance of dietary protein in supporting growth through nutrient sensing and signaling mechanisms [64,65].

The findings underscore the complex interplay between dietary protein levels and gene expression patterns related to immunity and growth in hybrid sturgeon, suggesting an optimal protein range for maximizing health and growth performance. However, it is important to acknowledge that while amino acids are known to influence gene expression, this does not necessarily translate directly to protein activity levels. Therefore, further research into the specific mechanisms by which dietary protein modulates these pathways, including studies on protein activity, will be critical for developing tailored nutritional strategies for aquaculture species. Such strategies aim to enhance productivity and disease resistance by bridging the gap between gene expression changes and actual protein functionality.

## 5. Conclusions

The study concludes that the optimal dietary protein level for juvenile hybrid sturgeon (*Acipenser baerii* × *A. schrenckii*) enhances growth, serum biochemistry, amino acid composition, and particularly influences intestine gene expression related to growth and immune functions. Key findings include a dietary protein range of 35.9% to 38.3% being optimal for growth, with significant effects on immune parameters such as lysozyme, complement components, immunoglobulin levels, and gene expression, including MyD88, TLRs, and TOR signaling pathways, demonstrating the intertwined relationship between nutrition, growth, and immune health in hybrid sturgeon. This finding is significant for the development of nutritionally balanced feeds, aiding in the efficient and semi-intensive culturing of hybrid sturgeon.

## Figures and Tables

**Table 1:** Composition and nutrient levels of proximate analysis (air-dried basis, %).

Items	Dietary Protein Levels					
	30% (G1)	33% (G2)	36% (G3)	39% (G4)	42% (G5)	45% (G6)
Casein	11.60	14.99	18.37	21.75	25.13	28.51
Dextrin	32.40	29.01	25.63	22.25	18.87	15.49
Fish meal	20.00	20.00	20.00	20.00	20.00	20.00
Wheat middlings	20.00	20.00	20.00	20.00	20.00	20.00
Wheat gluten	6.00	6.00	6.00	6.00	6.00	6.00
Fish oil	4.00	4.00	4.00	4.00	4.00	4.00
Soybean oil	4.00	4.00	4.00	4.00	4.00	4.00
Phospholipids	1.00	1.00	1.00	1.00	1.00	1.00
Premix	1.00	1.00	1.00	1.00	1.00	1.00
Nutrient levels						
Moisture (%)	8.53	8.43	8.31	8.41	8.32	8.74
Crude protein (%)	30.12	33.41	36.24	39.43	42.15	45.08
Crude lipid (%)	10.18	10.22	10.24	10.26	10.29	10.33
Nitrogen-free extract (%)	45.29	41.92	38.54	35.16	31.77	28.39
Gross energy (kJ/kg)	18.80	18.95	19.09	19.23	19.38	19.52
Essential amino acids						
Lysine	1.98	2.23	2.48	2.73	2.98	3.22
Methionine	0.67	0.77	0.86	0.95	1.04	1.13
Leucine	2.21	2.50	2.80	3.10	3.40	3.69
Isoleucine	1.19	1.35	1.50	1.66	1.82	1.98
Phenylalanine	1.21	1.37	1.53	1.69	1.85	2.02
Tyrosine	2.23	2.56	2.88	3.20	3.53	3.85
Tryptophan	0.32	0.36	0.40	0.43	0.47	0.51
Histidine	0.76	0.86	0.95	1.05	1.14	1.24
Valine	1.46	1.67	1.87	2.08	2.29	2.49
Threonine	1.11	1.24	1.38	1.51	1.65	1.78

Note: Premix 1.0%: choline 0.2%; antimildew 0.02%; magnesia 0.2%; zeolite 0.08%; vitamin 0.3%; mineral mixture 0.2%. Vitamin mixture (mg/kg): vitamin A 15,000 IU; vitamin B_1_ 15 mg; vitamin B_2_ 30 mg; vitamin B_6_ 15 mg; vitamin B_12_ 0.5 mg; vitamin C 1000 mg; vitamin D_3_ 3000 IU; vitamin E 60 mg; vitamin K_3_ 5 mg; nicotinic acid 175 mg; folic acid 5 mg; inositol 1000 mg; biotin 2.5 mg; pantothenic acid 50 mg. Mineral mixture (mg/kg): ZnSO_4_·7H_2_O 0.15 g, FeSO_4_·7H_2_O 0.10 g, MnSO_4_·4H_2_O 0.10 g, CuSO_4_·5H_2_O 0.02 g, CoCl_2_ 5 mg, KI 3 mg, Na_2_SeO_3_ 3 mg.

**Table 2:** Chemical analysis kits used in the experiment.

Items	Product Number	Method
SOD	A001-3	Based on the scavenging effect of SOD on superoxide radicals, one may gauge the activity of SOD indirectly through the measurement of the absorbance of the purple product (at 450 nm).
MDA	A003-1	MDA present in degradation products of lipid peroxides can react with thiobarbituric acid (TBA), and the amount of MDA can be determined by measuring the absorbance of the resulting red product (532 nm).
ACP	A059-2-2	Alkaline phosphatase activity can be determined by measuring the phenol produced when alkaline phosphatase hydrolyzes phenyl phosphate disodium substrate under alkaline conditions. The phenol reacts with 4-aminoantipyrine in the presence of potassium ferricyanide to form a red quinone complex that is measured at 520 nm.
LZM	A050-1-1	At a specific concentration of opaque bacterial solution, the lysozyme hydrolyses the peptidoglycan on the bacterial cell wall, resulting in bacterial cleavage and reduced concentration. This leads to an increase in transmittance, which is measured at 530 nm to determine the lysozyme content.
IgM	H109-1-1	The IgM level can be determined by measuring the turbidity produced when IgM in the sample forms an immune complex with anti-IgM antibodies in the reagents. After incubation with the reagents at 37 °C, the absorbance is measured at 340 nm, and the concentration is calculated from a nonlinear calibration curve.

Note: SOD, superoxide dismutase; MDA, malondialdehyde; ACP, alkaline phosphatase; LZM, lysozyme; IgM, immunoglobulin M.

**Table 3:** Primers used for determining gene expression of hybrid sturgeon.

Genes	Forward Primer Sequences (5′–3′)	Reverse Primer Sequences (5′–3′)	Length (bp)	Accession Number
MyD88	CACATGCGTCACTGTCAAGG	AGCATCACCAGCGAACTCAT	85	KU238084.1
GH	AACTCCCCGTCAGCATTCTG	AAGCAGCTCCACGTCTGATC	90	JX003684.1
IGF-1	TTCCGTCTTCCATCAGTGGC	AGTGTCCACAAGCTCAGCTC	154	FJ428828.1
TLR1	CCAGCAATGCATTTTCTGACCGTGT	AGTGAGTTGGCGCTGACATCCA	157	XM_034911210.1
TLR2	CTTTGCCTTCACAAACGCGA	CACTGCGAACAAAGTGCTCC	118	XM_034014252.2
IL-8	CATCCATCCCAGGCAGATC	TTGACCCAGCGGGCAGTT	112	MK140599.1
IL-6	TATACCAGCGGGAAGGACGA	GCTGCTGTGCGAGAGGATAT	141	XM_033993799.2
NF-κB	GCACAGCCTGGTTGGAAAG	AGACGCCGAAGTTGTAGCC	179	XM_034013617.2
IL-1β	GTGTGTGATGCTGGAGGTGA	GGCTCAGAGTCACTTGCTGT	197	MF818014.1
TNF-α	AGGAGCGGTCTCTACTTCGT	TGTGCGACAGATATACGGGC	82	XM_034909934.1
IL-10	CTACGGCAGTGTCGAAGTGT	TTGGGGTTGTGGAGTGCTTT	189	AY887900.1
TOR	GCCCAGCTTTCGCATATTGG	CGCTCGATCTCACCAGAGAC	95	OV754630.1
S6K1	TTTCGGACGAGGCCAAATCT	CACCTCTACACCTGCACACT	143	XM_059025314.1
4EBP1	GGAGTAACCATGTTTAACGCAGT	GACGCTCAGCAGCAACTTAC	247	XM_034052958.3
β-actin	GTTGTTGACAACGGTTCCGG	TCCTTCTGTCCCATGCCAAC	128	XM_034052523.2

Note: MyD88, myeloid differentiation primary response 88; GH, growth hormone; IGF-1, insulin-like growth factor 1; TLR1, Toll-like receptor 1; TLR2, Toll-like receptor 2; IL-8, interleukin 8; IL-6, interleukin 6; NF-κB, nuclear factor kappa-light-chain enhancer of activated B cells; IL-1β, interleukin 1 beta; TNF-α, tumor necrosis factor alpha; IL-10, interleukin 10; TOR, target of rapamycin; S6K1, S6 kinase 1; 4EBP1, eukaryotic translation initiation factor 4E binding protein 1.

**Table 4:** Real-time PCR amplification procedure.

Items	PCR Reaction Solution Preparation			PCR Amplification Procedure	
	Reagent	Consumption	Concentration	Procedure	Instrument
RT-PCR	TB Green Premix Ex Taq II (Tli RNaseH Plus)	10 µL	2×	Step 1: Reps: 1 95 °C 30 s Step 2: Reps: 40 95 °C 5 s 60 °C 34 s	7500 Real-Time PCR System; Applied Biosystems, Waltham, MA, USA
	ROX Reference Dye II	0.4 µL	50×		
	PCR Forward Primer	0.8 µL	10 µM		
	PCR Reverse Primer	0.8 µL	10 µM		
	cDNA	2 µL	50 ng/µL		
	DEPC H_2_O	6 µL			

**Table 5:** The growth performance of hybrid sturgeon fed diets containing different protein levels.

Items	Dietary Protein Levels					
	30% (G1)	33% (G2)	36% (G3)	39% (G4)	42% (G5)	45% (G6)
IBW (g)	29.70 ± 0.36	29.17 ± 0.50	30.47 ± 1.50	30.60 ± 0.9	29.63 ± 0.9	30.23 ± 1.4
FBW (g)	136.13 ± 12.12 ^a^	178.97 ± 25.81 ^b^	191.16 ± 13.61 ^b^	169.84 ± 11.47 ^ab^	177.16 ± 22.93 ^b^	158.09 ± 19.01 ^ab^
SR (%)	81.33 ± 7.02	86.01 ± 5.30	86.67 ± 5.03	88.00 ± 5.30	92.00 ± 8.71	89.33 ± 4.61
WGR (%)	358.07 ± 35.81 ^a^	514.55 ± 97.92 ^b^	529.32 ± 66.19 ^b^	512.74 ± 25.91 ^b^	499.79 ± 95.86 ^b^	422.89 ± 57.66 ^a^
SGR (%/d)	7.60 ± 0.40 ^a^	8.70 ± 0.32 ^b^	9.18 ± 0.55 ^b^	8.56 ± 0.23 ^ab^	8.91 ± 0.80 ^b^	8.25 ± 0.57 ^ab^
FCR	2.06 ± 0.10 ^a^	1.67 ± 0.35 ^b^	1.48 ± 0.14 ^b^	1.49 ± 0.13 ^b^	1.51 ± 0.68 ^b^	1.51 ± 0.74 ^b^
PER (%)	1.88 ± 0.91 ^a^	2.75 ± 0.62 ^ab^	3.37 ± 0.29 ^b^	2.75 ± 0.08 ^ab^	3.24 ± 0.82 ^b^	3.20 ± 0.51 ^b^
HSI (%)	2.67 ± 1.03 ^bc^	2.71 ± 0.52 ^bc^	2.11 ± 0.58 ^ab^	3.75 ± 1.05 ^c^	1.81 ± 0.51 ^ab^	0.80 ± 0.55 ^a^

Note: Values represent means ± S.E. (*n* = 3). Values in the same row with different superscript letters are significantly different (*p* < 0.05). IBW, initial mean body weight; FBW, final mean body weight; SR, survival rate; WGR, weight gain rate; SGR, specific growth rate; FCR, feed conversion ratio; PER, protein efficiency ratio; HIS, hepatosomatic index.

**Table 6:** Body composition of hybrid sturgeon fed diets containing different protein levels (%).

Items	Dietary Protein Levels					
	30% (G1)	33% (G2)	36% (G3)	39% (G4)	42% (G5)	45% (G6)
Moisture	72.04 ± 1.67	72.14 ± 1.50	72.73 ± 1.57	72.71 ± 1.44	72.82 ± 1.03	71.46 ± 1.55
Crude protein	13.30 ± 0.89 ^a^	13.49 ± 0.66 ^ab^	13.45 ± 0.78 ^ab^	13.46 ± 0.89 ^ab^	13.85 ± 0.77 ^b^	13.71 ± 0.94 ^b^
Crude lipid	11.12 ± 1.41 ^a^	11.41 ± 1.68 ^a^	11.86 ± 3.34 ^ab^	13.31 ± 1.41 ^b^	13.31 ± 1.47 ^b^	12.61 ± 1.21 ^ab^
Ash	2.30 ± 0.87 ^a^	2.49 ± 0.65 ^bc^	2.44 ± 0.77 ^bc^	2.46 ± 0.88 ^bc^	2.38 ± 0.76 ^b^	2.27 ± 0.93 ^a^

Note: Values represent means ± S.E. (*n* = 3). Values in the same row with different superscript letters are significantly different (*p* < 0.05).

**Table 7:** The whole-body amino acid composition of hybrid sturgeon fed diets containing different protein levels (%).

Items	Dietary Protein Levels					
	30% (G1)	33% (G2)	36% (G3)	39% (G4)	42% (G5)	45% (G6)
Essential amino acids						
Methionine	3.48 ± 0.02	3.52 ± 0.12	3.53 ± 0.11	3.51 ± 0.17	3.58 ± 0.04	3.51 ± 0.06
Threonine	2.84 ± 0.18	2.82 ± 0.41	3.09 ± 0.27	3.04 ± 0.28	3.34 ± 0.05	3.39 ± 0.07
Valine	3.28 ± 0.02 ^a^	3.49 ± 0.12 ^a^	3.53 ± 0.11 ^b^	3.51 ± 0.17 ^b^	3.78 ± 0.04 ^c^	3.81 ± 0.06 ^c^
Leucine	5.79 ± 0.07 ^a^	6.10 ± 0.19 ^b^	6.15 ± 0.13 ^b^	6.19 ± 0.21 ^b^	6.46 ± 0.15 ^c^	6.51 ± 0.07 ^c^
Phenylalanine	2.82 ± 0.03 ^a^	3.00 ± 0.16 ^a^	3.08 ± 0.15 ^a^	3.03 ± 0.24 ^a^	3.36 ± 0.09 ^b^	3.39 ± 0.01 ^b^
Isoleucine	3.11 ± 0.01 ^a^	3.29 ± 0.11 ^b^	3.33 ± 0.09 ^b^	3.34 ± 0.13 ^b^	3.52 ± 0.04 ^c^	3.56 ± 0.04 ^c^
Lysine	5.89 ± 0.07 ^a^	6.37 ± 0.33 ^b^	6.49 ± 0.28 ^b^	6.46 ± 0.45 ^b^	7.03 ± 0.06 ^c^	7.04 ± 0.08 ^c^
Histidine	1.65 ± 0.01	1.71 ± 0.24	1.61 ± 0.14	1.62 ± 0.18	1.85 ± 0.03	1.84 ± 0.01
Arginine	4.32 ± 0.06	4.46 ± 0.23	4.34 ± 0.27	4.56 ± 0.09	4.39 ± 0.05	4.48 ± 0.12
Non-essential amino acids						
Proline	2.01 ± 0.06 ^a^	2.15 ± 0.10 ^b^	2.20 ± 0.11 ^b^	2.25 ± 0.09 ^b^	2.38 ± 0.01 ^c^	2.39 ± 0.06 ^c^
Aspartic acid	3.93 ± 0.15	4.25 ± 0.56	3.77 ± 0.19	4.04 ± 0.17	3.77 ± 0.03	3.77 ± 0.04
Serine	3.22 ± 0.12	2.73 ± 0.37	3.12 ± 0.21	3.25 ± 0.09	3.16 ± 0.10	3.26 ± 0.05
Glutamic acid	11.26 ± 0.21 ^a^	12.01 ± 0.67 ^b^	12.57 ± 0.45 ^bc^	12.39 ± 0.44 ^bc^	13.00 ± 0.24 ^c^	13.09 ± 0.08 ^c^
Glycine	4.58 ± 0.02	4.42 ± 1.15	3.81 ± 1.23	4.58 ± 1.13	3.33 ± 0.05	3.43 ± 0.10
Alanine	4.24 ± 0.05	4.24 ± 0.13	4.2 ± 0.17	4.34 ± 0.08	4.38 ± 0.05	4.38 ± 0.05
Cystine	0.97 ± 0.01	0.99 ± 0.07	0.97 ± 0.05	0.96 ± 0.02	1.04 ± 0.01	1.03 ± 0.02
Tyrosine	2.61 ± 0.01	2.66 ± 0.13	2.6 ± 0.06	2.58 ± 0.01	2.71 ± 0.01	2.71 ± 0.06

Note: Values represent means ± S.E. (*n* = 3). Values in the same row with different superscript letters are significantly different (*p* < 0.05).

**Table 8:** The serum indices of hybrid sturgeon fed diets containing different protein levels.

Items	Dietary Protein Levels					
	30% (G1)	33% (G2)	36% (G3)	39% (G4)	42% (G5)	45% (G6)
GLU (mmol/L)	4.61 ± 0.91 ^a^	5.36 ± 0.63 ^ab^	6.03 ± 0.76 ^b^	5.96 ± 0.72 ^b^	5.08 ± 1.12 ^ab^	5.28 ± 1.15 ^ab^
TP (g/L)	27.32 ± 6.46	30.76 ± 4.04	30.29 ± 3.05	27.08 ± 3.10	29.47 ± 5.09	29.29 ± 8.19
ALB (g/L)	11.13 ± 1.53	12.67 ± 1.49	11.72 ± 1.58	11.49 ± 1.32	11.52 ± 1.28	11.77 ± 2.51
ALT (U/mL)	7.11 ± 3.33 ^ab^	9.11 ± 4.46 ^b^	7.78 ± 3.23 ^ab^	7.56 ± 1.81 ^ab^	5.89 ± 2.03 ^a^	5.22 ± 2.33 ^a^
AST (U/mL)	554.41 ± 268.15	694.45 ± 177.91	693.31 ± 214.30	701.00 ± 181.52	504.46 ± 64.83	562.22 ± 146.44
TG (mmol/L)	5.90 ± 2.56 ^a^	6.29 ± 1.38 ^ab^	8.23 ± 2.36 ^b^	6.69 ± 1.97 ^ab^	6.36 ± 2.22 ^ab^	5.77 ± 2.16 ^a^
SOD (U/mL)	44.85 ± 2.15 ^a^	50.49 ± 1.52 ^ab^	54.45 ± 4.02 ^b^	84.63 ± 6.21 ^e^	81.34 ± 0.83 ^e^	66.21 ± 2.07 ^d^
MDA (nmol/mL)	4.17 ± 0.12 ^e^	2.32 ± 0.18 ^b^	2.34 ± 0.06 ^b^	2.05 ± 0.09 ^a^	2.70 ± 0.07 ^c^	2.92 ± 0.15 ^d^

Note: Values represent means ± S.E. (*n* = 3). Values in the same row with different superscript letters are significantly different (*p* < 0.05). GLU, glucose; TP, total protein; ALB, albumin; ALT, alanine aminotransferase; AST, aspartate aminotransferase; TG, triglyceride; SOD, superoxide dismutase; MDA, malondialdehyde.

**Table 9:** The serum immune and inflammatory markers of hybrid sturgeon fed diets containing different protein levels (%).

Items	Dietary Protein Levels					
	30% (G1)	33% (G2)	36% (G3)	39% (G4)	42% (G5)	45% (G6)
LZM (U/mL)	99.46 ± 10.34 ^a^	190.48 ± 18.47 ^b^	247.96 ± 19.07 ^d^	251.25 ± 18.90 ^d^	287.53 ± 11.99 ^e^	218.24 ± 17.06 ^c^
ACP (U/mL)	257.97 ± 6.57 ^a^	273.77 ± 7.19 ^b^	326.82 ± 4.74 ^d^	370.82 ± 3.95 ^e^	365.21 ± 4.23 ^e^	287.14 ± 4.63 ^c^
C3 (mg/mL)	12.38 ± 1.15 ^a^	14.17 ± 1.41 ^b^	16.41 ± 0.56 ^d^	15.68 ± 0.55 ^cd^	14.83 ± 1.41 ^bc^	12.66 ± 1.08 ^a^
C4 (mg/mL)	1.39 ± 0.15 ^a^	1.52 ± 0.13 ^b^	1.66 ± 0.18 ^c^	1.74 ± 0.20 ^cd^	1.75 ± 0.16 ^d^	1.70 ± 0.16 ^cd^
IgM (mg/mL)	94.92 ± 3.84 ^a^	109.18 ± 9.41 ^b^	118.90 ± 8.55 ^c^	113.28 ± 4.22 ^bc^	112.73 ± 6.42 ^bc^	113.11 ± 7.19 ^bc^

Note: Value represents mean ± S.E. (*n* = 3). Values in the same row with different superscript letters are significantly different (*p* < 0.05). LZM, lysozyme; ACP, acid phosphatase; C3, complement 3; C4, complement 4; IgM, globulin M.

## Data Availability

The original contributions presented in the study are included in the article, and further inquiries can be directed to the corresponding authors.
